# Pharmacokinetics and Tissue Distribution Study of Caudatin in Normal and Diethylnitrosamine-Induced Hepatocellular Carcinoma Model Rats

**DOI:** 10.3390/molecules20034225

**Published:** 2015-03-05

**Authors:** Yunru Peng, Yongfang Ding

**Affiliations:** Department of Pharmacology and Toxicology, Jiangsu Provincial Institute of Traditional Chinese Medicine, 100 Shizi Street, Nanjing 210028, China; E-Mail: angelding9@hotmail.com

**Keywords:** pharmacokinetics, C-21 steroidal glycoside, *Cynanchum auriculatum* Royle ex wight, caudatin, hepatocellular carcinoma

## Abstract

Caudatin is a potential antitumor agent isolated from the traditional Chinese medicine “*baishouwu*”, which was the root tuber of *Cynanchum auriculatum* Royle ex Wight. In our previous studies, caudatin showed selectivity on human hepatoma cell line SMMC7721 among several different tumor cell lines, and further *in vivo* tests validated the inhibitory action of caudatin against hepatic cancer using an H_22_ solid tumor model in mice, but to our knowledge, the biopharmaceutical properties of caudatin are largely unknown. In this study, a simple, rapid and sensitive ultra-performance liquid chromatography-tandem mass spectrometry (UPLC-MS/MS) method for the determination of caudatin in rat plasma and tissues, which kept the run time to detect one sample within 4 min, was developed and validated. Pharmacokinetics and tissue distribution studies of caudatin in conventional rats and hepatocellular carcinoma (HCC) model rats were then conducted for the first time. Statistically significant differences were observed between conventional rats and diethylnitrosamine (DEN)-induced HCC rats with respect to pharmacokinetic parameters, including maximum concentration (C_max_), time to reach C_max_ (*T*_max_), half-life (*t*_1/2_), area under the concentration-time curve (AUC_0-t_, AUC_0-∞_), mean residence time (MRT_0-t_ and MRT_0-∞_), and oral clearance (CL/F). Increased exposures of caudatin were found in the plasma and livers of HCC model rats, which would be helpful for a better understanding of pharmacological effect of caudatin in treating HCC disease.

## 1. Introduction

Hepatocellular carcinoma (HCC) is one of the most common cancers, associated with high mortality, and the world’s highest morbidity and mortality rates for this malignant tumor are found in China [1]. Since HCC mostly arises from chronic hepatitis and liver cirrhosis caused by infection with hepatitis C virus (HCV) or hepatitis B virus (HBV), novel antihepatocarcinogenic agents exhibiting comprehensive effects in therapy of chronic hepatitis and prevention liver cirrhosis are required [2].

The root of *Cynanchum auriculatum* Royle ex Wight, known as “*baishouwu*” in China, has been widely used in the clinic as a beneficial and tonic agent for long times. Its major active components, C-21 steroidal glycosides, are of considerable interest because of their bioactivities, including prevention and therapy of chronic hepatitis [3], hepatic fibrosis [4] and liver cancer [5,6,7]. In our previous studies, several C-21 steroidal glycosides were isolated from *baishouwu*, of which caudatin was the most potent compound against tumor cell lines and showed selectivity on human hepatoma cell line SMMC7721 among several different tumor cell lines [8]. On account of the results, we speculated that the C-21 steroidal glycosides obtained in our work were sensitive to some human tumor cell lines such as liver cancer cell lines. The *in vivo* assays further showed that caudatin significantly inhibited the growth of transplantable H_22_ tumors in mice [8]. Further *in vitro* studies illustrated that the anticancer activity of caudatin could be attributed partly to its inhibition of cell proliferation and induction of apoptosis in human hepatoma cell line SMMC7721 through caspase activation [9]. Recent studies also proved that caudatin could induce cell cycle arrest and caspase-dependent apoptosis in HepG2 cells [10]. Interestingly, caudatin derivatives were recently reported to exhibit potent anti-HBV activity and could be developed as novel anti-HBV agents [11,12], from which we hypothesized that the mechanism of caudatin for treating hepatocellular carcinoma was comprehensive. As a potential inhibitor of liver cancer, the biopharmaceutical properties of caudatin are largely unknown, so in our previous study an oral pharmacokinetic evaluation of caudatin in rats was conducted, which revealed a non-linear profile between 10 and 40 mg/kg [13]. Up to now, the pharmacokinetics of caudatin in hepatocellular carcinoma model animals remains unclear. Considering the anti-hepatocarcinogenic effect of caudatin, it is important to compare the differences of its pharmacokinetics and distribution in conventional and hepatocellular carcinoma model animals.

Therefore, in the present study a comparative investigation of the pharmacokinetics and tissue distribution of caudatin in conventional and DEN-induced hepatocellular carcinoma model rats was conducted. In our previous studies, a novel liquid chromatography/electrospray ionization-tandem mass spectrometry (LC/ESI-MS/MS) method in positive multiple reaction monitoring (MRM) mode was developed for the determination of caudatin in rat plasma [14]. Owing to the progress of the apparatus, a more efficient, sensitive rapid resolution liquid chromatography/tandem mass spectrometry (UPLC–MS/MS) method for the determination of caudatin in rat plasma and different tissues had been developed and validated in this study, which purpose was to evaluate the pharmacokinetics and tissue distribution of caudatin in conventional and DEN-induced hepatocellular carcinoma model rats. To the best of our knowledge, no studies have provided unequivocal evidence on the pharmacokinetic properties of caudatin in hepatocellular carcinoma model rats.

## 2. Results and Discussion

### 2.1. Method Development

In this study, mass spectrometry (MS) detecting conditions were operated according to the MS signal response of the target compound and the results indicated that the negative mode was much more sensitive than the positive mode for both caudatin and the internal standard (IS). In order to achieve good resolution, symmetric peak shapes for caudatin and IS and a short run time, the chromatographic conditions were optimized through several trials. Finally, a Waters ACQUITY^TM^ UPLC^TM^ BEH C18 column (100 × 2.1 mm, 1.7 μm) was used for the chromatographic separation. It was found that a mixture of 0.1% formic acid and acetonitrile could achieve our purpose and was finally adopted as the mobile phase for the chromatographic separation.

### 2.2. Method Validation

**Figure 1:**
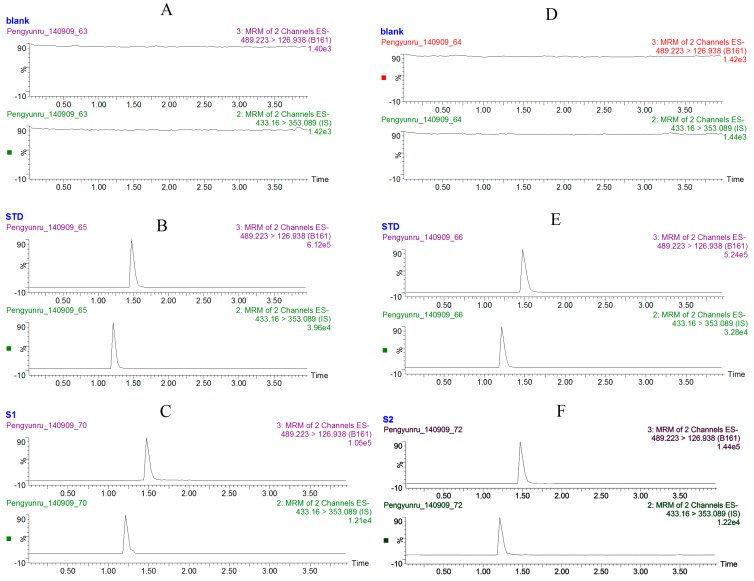
Typical chromatograms of caudatin. (**A**) blank plasma; (**B**) blank plasma spiked with caudatin and IS; (**C**) 0.5 h plasma sample after administration of caudatin at a dose of 20 mg/kg; (**D**) blank liver tissue homogenate; (**E**) blank liver tissue homogenate spiked with caudatin and IS; (**F**) 0.5 h liver homogenate sample after administration of caudatin at a dose of 20 mg/kg.

The detection of caudatin and IS by MRM was highly selective with no interference. Typical chromatograms of blank plasma or tissue homogenate, blank plasma or tissue homogenate spiked with caudatin and IS, samples from rats after oral administration at a dose of 20 mg/kg are presented in Figure 1. The retention times were 1.49 min for caudatin, 1.26 min for IS. The MS spectra of caudatin and IS were monitored in negative ion mode and [M−H]^−^ ions were observed as the predominant ions for both of them.

Standard curves prepared for caudatin in plasma and tissue homogenates showed good linearity over the concentration ranges of 5–1000 ng/mL and the lower limit of quantification was 5 ng/mL. The lower limit of detection was 1.2 ng/mL.

The accuracy and precision of the method were evaluated based on the data from the quality control (QC) plasma and liver tissue homogenate samples at three concentrations (10, 100 and 800 ng/mL) in three validation runs. As shown in Table 1, the results of the tested samples were all within the acceptable criteria (relative standard deviation (RSD)% <15%; accuracy error (RE)% less than ±15%), indicating the acceptable accuracy and precision of the method developed.

The extraction recoveries of caudatin from rat plasma and tissue homogenate samples were all more than 75.0% at different concentration levels (Table 1) and the extraction recovery of IS was more than 75.0%, which indicated that the recoveries of caudatin and IS were consistent, precise and reproducible at different concentration levels.

The possibility of matrix effect caused by ionization competition between the analytes and the endogenous co-eluents was evaluated at three concentrations in six replicates. No significant matrix effect was observed for the analyte in the samples.

The measured concentration of caudatin at each QC level deviated within 15.0%, which demonstrated that they were stable in plasma and tissue homogenate samples at room temperature for 48 h, at −20 °C for at least 30 days and after three freeze and thaw cyles (Table 2).

**Table 1:** Accuracy, precision and recovery of caudatin in rat plasma and tissue homogenate (n = 5).

	Concentration (ng/mL)	Intra-day R.S.D. (%)	Inter-day R.S.D. (%)	Accuracy R.E. (%)	Recovery (%)
plasma	10	9.5	9.2	6.6	95.6
	100	6.8	8.4	−5.5	96.8
	800	7.6	6.5	8.7	92.4
tissue homogenate	10	6.9	7.7	−8.9	99.3
	100	8.7	9.4	7.1	91.1
	800	8.6	9.5	5.5	93.1

### 2.3. Method Application

#### 2.3.1. Pharmacokinetic Study

**Figure 2:**
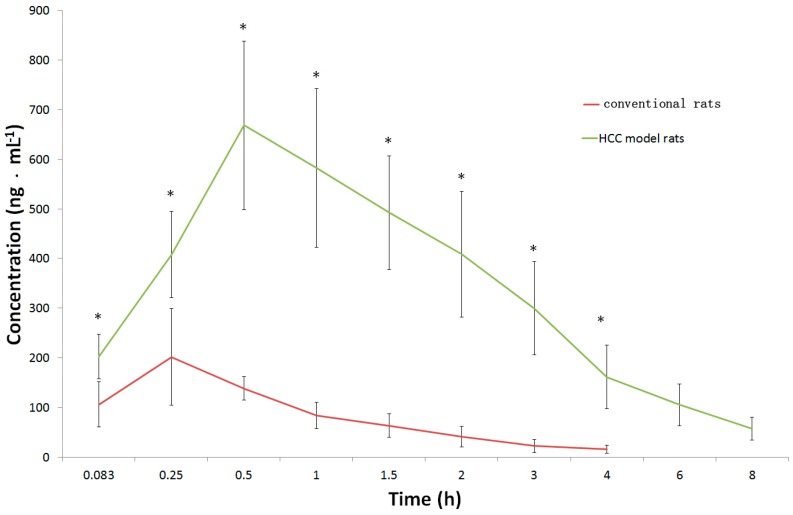
Mean plasma concentration-time profiles of caudatin after oral administration at a dose of 20 mg/kg in conventional and HCC rats. Significantly different from the conventional rats (*, *p* < 0.05).

Pharmacokinetic studies were performed in conventional rats and DEN-induced HCC rats after a single dose of caudatin at 20 mg/kg to show the applicability of the validated UPLC-MS/MS method. The mean plasma concentration-time profiles were shown in Figure 2. The selected pharmacokinetic parameters are listed in Table 3.

**Table 2:** Stability of caudatin in rat plasma and tissue homogenate (n = 5).

	Stability	Added C (ng/mL)	Found C (ng/mL)	Accuracy R.E. (%)
plasma	48 h at room temperature	10	9.1	−9.0
		100	95.7	−4.3
		800	789.4	−1.3
	1 month at −20 °C	10	9.6	−4.0
		100	96.2	−3.8
		800	814.2	1.8
	three freeze-thaw cycles	10	10.4	4.0
		100	94.2	−5.8
		800	759.1	−5.1
tissue homogenate	48 h at room temperature	10	10.3	3.0
		100	92.3	−7.7
		800	745.5	−6.8
	1 month at −20 °C	10	9.2	−8.0
		100	93.2	−6.8
		800	736.6	−7.9
	three freeze–thaw cycles	10	9.4	−6.0
		100	105.4	5.4
		800	857.3	7.2

**Table 3:** Pharmacokinetic parameters of caudatin in conventional and HCC model rats after oral administration at a dose of 20 mg/kg (n = 6).

Parameter	Unit	Conventional Rats	HCC Model Rats
AUC(0-t)	µg·h/L	236.8 ± 62.8	2006.0 ± 334.1 *
AUC(0-∞)	µg·h/L	263.4 ± 76.2	2251.9 ± 386.0 *
MRT(0-t)	h	1.21 ± 0.13	2.47 ± 0.32 *
MRT(0-∞)	h	1.68 ± 0.39	3.62 ± 1.11 *
T_1/2z_	h	1.25 ± 0.49	2.89 ± 1.78 *
T_max_	h	0.29 ± 0.10	1.00 ± 0.45 *
C_max_	µg/L	314.7 ± 82.0	713.4 ± 129.3 *
CLz/F	L/h/kg	80.8 ± 20.8	9.13 ± 1.75 *
Vz/F	L/kg	147.7 ± 78.0	37.2 ± 21.2 *

* *p* < 0.05, compared with the conventional rats.

Caudatin was rapidly absorbed in conventional rats, and its high concentration level could only be detected at 0.29 ± 0.10 h after oral administration. The plasma maximum concentration (C_max_) and area under the concentration-time curve (AUC_0-t_) of caudatin after oral administration to conventional rats were 314.7 ± 82.0 µg/L and 236.8 ± 62.8 µg·h/L, respectively. Statistically significant differences were observed between conventional rats and DEN-induced HCC rats with respect to pharmacokinetic parameters including C_max_, *T*_max_, *t*_1/2_, AUC_0-t_, AUC_0-∞_, mean residence time (MRT_0-t_ and MRT_0-∞_), and oral clearance (CL/F). Compared with conventional rats, the HCC conditions induced 2.31-, 3.45-, 2.27- and 8.47-fold increases in elimination half-life (*t*_1/2_), *T*_max_, C_max_ and AUC_0-t_ for caudatin, respectively. Vz/F and CL/F values for caudatin in conventional rats were increased 3.97- and 8.85-fold, compared with the corresponding parameters observed in HCC rats. The result indicated that caudatin was eliminated rapidly in blood circulation in conventional rats. The HCC conditions could remarkably increase the blood concentration of caudain and prolong its retention in blood.

In the present study, large differences in all measured PK parameters for caudatin between the conventional and HCC model rats were found and exposure of caudatin in the plasma of HCC rats was increased obviously with C_max_ and AUC values being increased 2.27- and 8.47-fold, respectively, compared with those in conventional rats. This difference in oral bioavailability of caudatin in conventional and HCC model rats may due to some reason such as drug metabolism. Several reports have indicated that the enzymes involved in phase I and phase II metabolism underwent apparent changes in patients with hepatocellular carcinoma [15,16,17,18], which could impact drug metabolism, disposition and pharmacotherapy. Moreover, animal experiments also proved that there were obvious differences in drug metabolism between the normal rats and the diethylnitrosamine-induced HCC model rats [19,20]. Perhaps we could presume that the changes in the metabolism in HCC rats might partially explain the different pharmacokinetic behaviors of caudatin among normal and model rats. Further studies are needed to better understand the mechanism of this difference.

#### 2.3.2. Tissue Distribution Study

**Figure 3:**
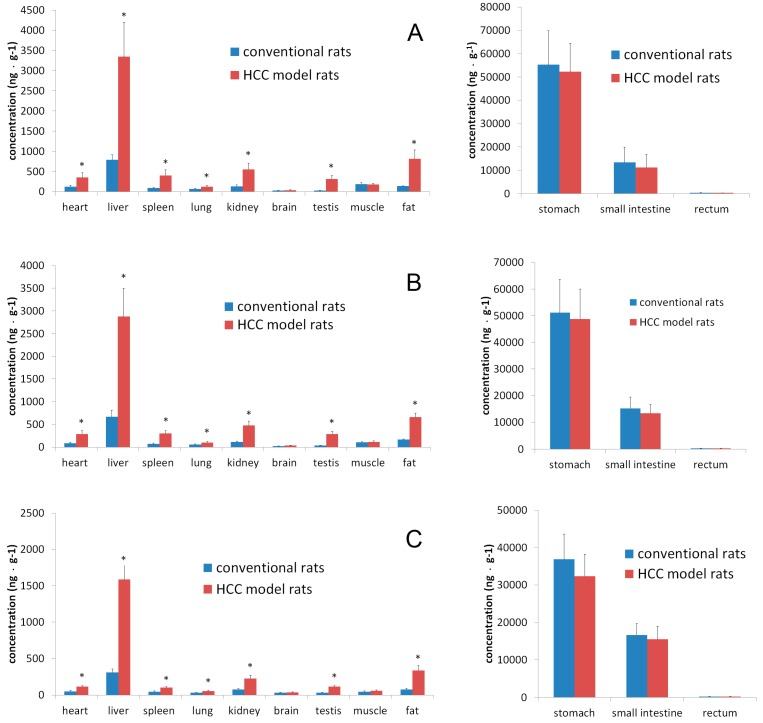
The concentration of caudatin in tissues after oral administration at a dose of 20 mg/kg in conventional and HCC rats. (**A**) 0.5 h; (**B**) 1.0 h; (**C**) 3.0 h. Significantly different from the conventional rats (*, *p* < 0.05).

The profile of caudatin in tissue homogenate (heart, liver, spleen, lung, kidney, brain, stomach, small intestine, rectum, testis, muscle and fat) after oral administration of caudatin at 0.5 h, 1.0 h and 3.0 h is shown in Figure 3. According to the time process of distribution, caudatin was observed in most tissues just 0.5 h after oral administration. At this moment, caudatin was concentrated in the digestive system, especially in the stomach and small intestine, which showed the main tissue absorption. Compared with conventional rats, the HCC conditions significantly increased concentrations of caudatin in heart, liver, spleen, kidney, testis and fat, especially in liver (Table 4). The concentrations of caudatin found in livers exceeded those observed in other tissues at three sampling-time points, aside from the digestive system. The result revealed significant tissue targeting of caudatin after oral administration.

**Table 4:** Fold increase in plasma or tissues of caudatin in HCC model rats *vs* conventional rats (n = 5).

Plasma or Tissues	Fold Increase		
	0.5 h	1.0 h	3.0 h
plasma	4.83	6.95	13.36
heart	2.91	3.37	2.23
liver	4.26	4.30	5.10
spleen	4.63	4.26	2.31
lung	1.83	1.82	1.65
kidney	4.18	4.19	3.05
brain	1.48	1.56	1.05
testis	13.89	7.90	3.38
muscle	0.94	1.09	1.30
fat	5.95	3.96	4.46
stomach	0.95	0.95	0.88
small intestine	0.84	0.88	0.93
rectum	0.97	1.06	0.93

## 3. Experimental

### 3.1. Materials, Reagents and Animals

**Figure 4:**
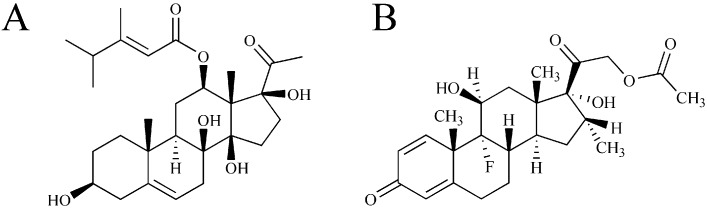
The chemical structures of caudatin (**A**) and dexamethasone acetate (**B**).

Caudatin (98.6% purity) was isolated from the root tubers of *C. auriculatum* according to the previously reported protocols [8]. The internal standard, dexamethasone acetate (99.2% purity, 100122-201206), was obtained from the National Institute for the Control of Pharmaceutical and Biological Products (Beijing, China). The structures of caudatin and IS are displayed in Figure 4. HPLC grade acetonitrile and methanol were purchased from Merck (Darmstadt, Germany). Deionized water was purified by a Milli-Q system (Millipore, Bedford, MA, USA). All the other reagents were of analytical grade.

Male Sprague-Dawley rats (180–200 g) were provided by Shanghai SLAC Laboratory Animal Co. (Shanghai, China). Animals were maintained in an environmentally controlled room under controlled temperature (22–24 °C) and relative humidity (40%–70%) with a 12-h light/dark cycle. The animal care, use, and experimental protocols were approved by the animal care committee of Jiangsu Provincial Institute of Traditional Chinese Medicine.

**Figure 5:**
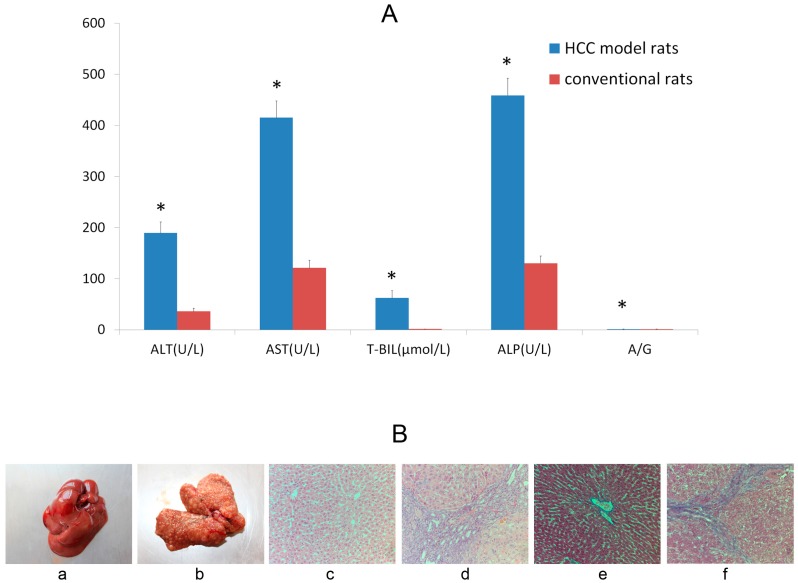
The represent of biochemistry parameters and histological evaluation in HCC model rats. (**A**) Biochemistry parameters of liver function; Significantly different from the conventional rats (*, *p* < 0.05). (**B**) Representative photographs. Macroscopic findings in liver of conventional (**a**) and HCC model rats (**b**). Histopathologic examination (HE staining, ×200) in liver of conventional (**c**) and HCC model rats (**d**). Masson staining (×200) for liver of conventional (**e**) and HCC model rats (**f**).

Fifty-two male Sprague-Dawley rats were randomly divided into hepatocellular carcinoma (HCC) model group (n = 30) and normal control group (n = 22). Diethylnitrosamine (10 mg/kg) was orally administered to rats of model group five times a week for 14 weeks, and the normal control group received vehicle [21]. At the end of 16th week, blood samples were collected via the angular vein for liver function test for all animals and one rat in each group was randomly sacrificed and liver tissue was stained with hematoxylin-eosin (HE) and Masson for histopathologic examination (Figure 5). Macroscopic and microscopic features of the liver were evaluated, which showed that the HCC model of rats had been established. The experiments were performed at the end of 16th week since administration.

### 3.2. Instrumentation and Conditions

The analysis of plasma and tissue samples was conducted on a Waters Acquity UPLC system (Waters, Milford, MA, USA), consisting of a binary solvent delivery system and an autosampler. UPLC separation was achieved on a Waters ACQUITY^TM^ UPLC^TM^ BEH C18 column (100 × 2.1 mm, 1.7 μm) with gradient elution of 0.10% formic acid (A) and acetonitrile (B) at a flow rate of 0.4 mL/min, and column temperature 35 °C. The gradient program was as follows: 0–2 min, 50%–95% B; 2–3 min, 95%–95% B. The injection volume was 2 μL and the total time needed to analyze one sample only is 4 min. All the analyses were operated using MassLynx XS Software. A Waters Corp. eXevo Triple Quadrupole MS equipped with an electrospray ionization (ESI) source was used for mass spectrometry detection. The parameters in the source were set as follows: capillary voltage, 3.0 kV; desolvation gas rate, 1,000 L/h; desolvation temperature, 500 °C; cone gas flow rate, 50 L/h; source temperature 150 °C. The analyte detection was performed by using the multiple reaction monitoring (MRM) technique.

### 3.3. Standard Solution and Quality Control Samples

The stock solution of caudatin was prepared by dissolving 10.0 mg accurately weighed standard compound in 10.0 mL methanol to produce a concentration of 1.0 mg/mL, and was stored at 4 °C. A series of working solutions of caudatin were prepared freshly in methanol by diluting the stock solution in appropriate ratios at seven concentrations of 5–1000 ng/mL for plasma or tissue samples. A stock solution of dexamethasone acetate (1 mg/mL) was prepared in methanol, from which a 4000 ng/mL internal standard (IS) working solution was prepared in methanol as well and stored at 4 °C. Three concentration levels of quality control samples (QCs) were prepared containing caudatin (10, 100 and 800 ng/mL) and IS (400 ng/mL) in the same manner.

### 3.4. Sample Preparation

All samples, QCs, and standards with a sample volume of 90 μL spiked with 10 μL of IS working solution were extracted with 1.5 mL of ethyl acetate. The mixture was vortexed for 2 min and centrifuged at 4000 r/min for 5 min. The supernatant was pipetted into a 2 mL glass test tube and evaporated at 60 °C under nitrogen. The residue was reconstituted with 150 µL of mobile phase and followed by vortexing for 1 min and centrifuged at 4000 r/min for 5 min. The samples were transferred into autosampler vials with inner bracing tubes for analysis, respectively.

### 3.5. Method Validation

The method was validated for accuracy, precision, selectivity, calibration curve range, and reproducibility over a concentration range of 5–1000 ng/mL using three calibration standards, and five replicates of QC samples at each concentration level in three separate runs. The lower limit of quantification (LLOQ) for caudatin in plasma was defined as the lowest concentration, linear with the calibration curves having a relative error (RE%) below 20% and accuracy between 80% and 120%. The lower limit of detection (LLOD on column) was defined as the amount that could be detected with a signal-to-noise ratio of 3.

The precision and accuracy of the entire method were assessed at three QC concentration levels (10, 100, 800 ng/mL), each extracted and analyzed in five replicates on the same day (intra-day precision and accuracy) and on three consecutive days (inter-day precision and accuracy). Precision was defined as the relative standard deviation (RSD) and accuracy was calculated as the relative difference between calculated and nominal concentration of the QC samples (bias). The intra- and inter-day precision and bias were set at ≤15%.

The extraction recoveries of caudatin were calculated by comparing the peak-area ratios (caudatin/IS) of extracted plasma standards to the peak-area ratios of post-extraction plasma blanks spiked at corresponding concentrations. The recovery experiments were performed with three QC concentrations (10, 100, 800 ng/mL), with five replicates at each concentration. The matrix effect was investigated by extracting “blank” normal plasma and reconstituting with methanol containing a known amount of the analytes, analyzing the reconstituted extracts, and then comparing the peak areas of the analytes with that of analytes in methanol.

Sample stability was determined by analyzing QC samples stored for 48 h at room temperature, 1 month at −20 °C and subjected to three freeze–thaw cycles. For each of the storage conditions, five replicates were analyzed at three concentration levels.

### 3.6. Application to Pharmacokinetics and Tissue Distributions

After the histopathologic examination showed the HCC model of rats had been established, 21 rats were selected randomly from HCC model group (six rats died in model development) for pharmacokinetic and distribution studies. The rats were divided into two groups: 21 conventional rats in group A and 21 DEN-induced HCC rats in group B, with six rats per group for blood withdrawn and 15 rats for getting tissue samples. The animals were deprived of food for 12 h with water before the experiment and received an oral administration at dose of 20 mg/kg. Blood samples (about 250 μL) were collected into heparinized Eppendorf tubes at 0, 0.083, 0.25, 0.5, 1, 1.5, 2, 3, 4, 6, 8 h after dosing. Then the blood samples were immediately centrifuged and kept frozen at −20 °C until analysis. Five rats were decapitated, tissues including the heart, liver, spleen, lung, kidney, brain, stomach, small intestine, rectum, testis, muscle and fat were removed, washed of residual blood, and weighed at 0.5 h, 1.0 h, 3.0 h after drug administration. Each tissue sample was homogenized with deionized water (1 g: 9 mL), centrifuged and stored at −20 °C until analysis. All pharmacokinetic parameters were processed by noncompartmental analysis using the DAS software. A two-tailed Student’s *t* test was used to compare the pharmacokinetic parameters between the conventional rats and model rats. *p* ≤0.05 was considered statistically significant.

## 4. Conclusions

A simple, rapid and sensitive UPLC-MS/MS method was developed and validated for the determination of caudatin in rat plasma and tissues, which kept the run time to detect one sample under 4 min. Pharmacokinetics and tissue distribution studies of caudatin in conventional rats and HCC model rats were conducted and the results indicated that the pharmacokinetic profiles and tissue distribution were statistically different. Increased exposures of caudatin were found in the plasma and livers of HCC model rats, which should be helpful for a better understanding of the pharmacological effect of caudatin in treating HCC disease.
